# Transcriptome analysis of embryonic muscle development in Chengkou Mountain Chicken

**DOI:** 10.1186/s12864-021-07740-w

**Published:** 2021-06-09

**Authors:** Lingtong Ren, Anfang Liu, Qigui Wang, Honggan Wang, Deqiang Dong, Lingbin Liu

**Affiliations:** 1 College of Animal Science and Technology, Southwest University, Beibei, 400715 Chongqing, P. R. China; 2 ChongQing Academy of Animal Sciences, Rongchang, 402460 Chongqing, P. R. China

**Keywords:** Chengkou Mountain Chicken, Embryo muscle development, Transcriptome analysis

## Abstract

**Background:**

Muscle is the predominant portion of any meat product, and growth performance and product quality are the core of modern breeding. The embryonic period is highly critical for muscle development, the number, shape and structure of muscle fibers are determined at the embryonic stage. Herein, we performed transcriptome analysis to reveal the law of muscle development in the embryonic stage of Chengkou Mountain Chicken at embryonic days (E) 12, 16, 19, 21.

**Results:**

Diameter and area of muscle fibers exhibited significant difference at different embryonic times(*P < 0.01*). A total of 16,330 mRNAs transcripts were detected, including 109 novel mRNAs transcripts. By comparing different embryonic muscle development time points, 2,262 in E12vsE16, 5,058 in E12vsE19, 6139 in E12vsE21, 1,282 in E16vsE19, 2,920 in E16vsE21, and 646 in E19vsE21differentially expressed mRNAs were identified. It is worth noting that 7,572 mRNAs were differentially expressed. The time-series expression profile of differentially expressed genes (DEGs) showed that the rising and falling expression trends were significantly enriched. The significant enrichment trends included 3,150 DEGs. GO enrichment analysis provided three significantly enriched categories of significantly enriched differential genes, including 65 cellular components, 88 molecular functions, and 453 biological processes. Through KEGG analysis, we explored the biological metabolic pathways involved in differentially expressed genes. A total of 177 KEGG pathways were enriched, including 19 significant pathways, such as extracellular matrix-receptor interactions. Similarly, numerous pathways related to muscle development were found, including the Wnt signaling pathway (*P < 0.05*), MAPK signalingpathway, TGF-beta signaling pathway, PI3K-Akt signaling pathway and mTOR signaling pathway. Among the differentially expressed genes, we selected those involved in developing 4-time points; notably, up-regulated genes included *MYH1F*, *SLC25A12*, and *HADHB*, whereas the down-regulated genes included *STMN1*, *VASH2*, and *TUBAL3*.

**Conclusions:**

Our study explored the embryonic muscle development of the Chengkou Mountain Chicken. A large number of DEGs related to muscle development have been identified ,and validation of key genes for embryonic development and preliminary explanation of their role in muscle development. Overall, this study broadened our current understanding of the phenotypic mechanism for myofiber formation and provides valuable information for improving chicken quality.

**Supplementary Information:**

The online version contains supplementary material available at 10.1186/s12864-021-07740-w.

## Background

Meat products are essential for human life. Chicken is the second largest category of meat products consumed in China after pork [1]. Due to the increasing need for a better life among the people, the fast-growing supply for livestock and poultry products cannot meet the demand. Therefore, improving the quality of meat products and maintaining a high growth rate has become the focus of research. Muscle development is generally classified into two stages, embryonic period and after birth [2]. In the embryonic stage, muscle progenitor cells undergo differentiation and proliferation to form myoblasts, which then fuse to form multinucleated myotubes. Finally, myotubes mature into myofibers with contractile properties [3]. Simultaneously, the deposition of many substances related to the flavor of meat products is initiated during the embryonic period. The postnatal muscle development depends on the myocyte proliferation and differentiation with the muscle satellite cell function exertion [4]. Among them, the myofiber morphological structure and quantity are completed in the embryonic period, highlighting the need to explore embryonic muscle development in poultry.

Myogenesis is a complex biological process involving a large number of gene regulatory networks [3, 5], such as myogenic regulatory factors (MRFs) [6], myocyte enhancer factor-2(MEF2), and Insulin-Like Growth Factors (IGFs) [7]. In most cases, there are interactions between genes, and how they participate in muscle development is continually being investigated. As a member of the MRFs family, *MyoD* is widely involved in myogenic differentiation [6, 8]. The knockdown or knockout of *MyoD* stalls muscle differentiation, impeding the muscle generation process [9]. At the same time, *MyoD* harbors multiple associated genes, including *Myf5, MEF2*, and *MRF4*, which regulate muscle generation and regeneration in the form of gene networks.

With the continuous progress of scientific research, the depth and breadth of sequencing have deepened, and the cost of sequencing has decreased. For instance, Omics technology is widely used in today’s livestock breeding. Using transcriptomics, gene expression can be elucidated at the transcriptional level, mainly using second-generation sequencing technology [10]. RNA-seq technology proved helpful in revealing the essential genes and pathways associated with muscle development. In previous studys, different growth rate chicken (Jinghai Yellow Chicken) muscle was used to analyze the expression difference of genes about muscle development, which revealed the regulation mechanism of the differently growth chickens [11, 12]. Transcriptome sequencing was performed on the breast muscle and leg muscle of Hanzhong Mabu ducks at several time points during embryo and postnatal period to find the key genes that play regulatory roles at different time points, and to provide a basis for further research on the growth and development mechanism of duck skeletal muscle[13].Zhao et al.[14] used longissimus dorsi muscle of Lantang and Landrace pig at different gestation times as the research object to explore the muscle development rules of pig embryos of different breeds. The selection of economic traits is currently the primary goal of poultry breeding, and investigations on the skeletal muscle molecular regulation have attracted immense research interest. People are gradually looking for nutritious, green, and healthy poultry breeds, and the Chengkou Mountain Chicken is highly considered. As a unique local chicken breed in Chongqing, China, it possesses the characteristics of typical mountain chickens in the southwestern mountainous area of China. It is characterized by resistance to rough feeding, strong adaptability, delicious meat products, and has high nutritional value [15]. Because of the importance of the embryonic stage in muscle development, we chose the Chengkou Mountain Chicken’s embryonic muscle as the materiale to explore the mechanism about muscle development. In the middle and late stages of poultry’s embryonic muscle development, breast muscles appeared to be slower than leg muscles. Therefore, we chose four stages (E12, E16, E19, E21) of leg muscles to perform transcriptome sequencing, to determine the unique gene expression pattern of local chicken breeds and provide a new theoretical basis in poultry breeding.

## Results

### Histological characteristics of muscle

**Fig. 1:**
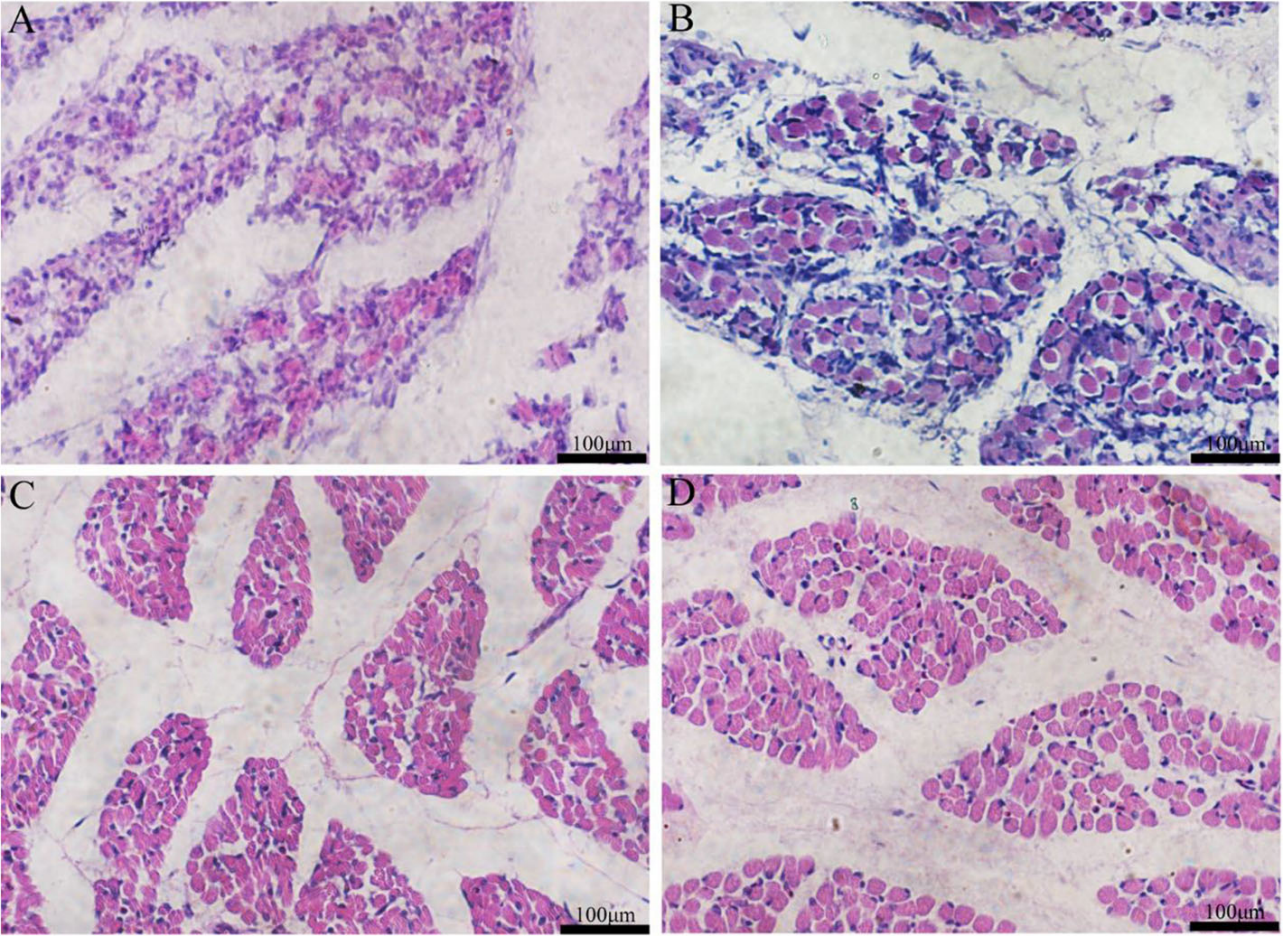
Embryo muscle histological observation. Histological characteristics in E12 (**A**) (Intact muscle fibers were not formed), E16 (**B**), E19(**C**), E21 (**D**). (scaleplate:100 μm)

To assess the muscle development regulation in the embryo of the Chengkou Mountain Chicken, we obtained embryonal muscle data at multiple time points. Muscle fibers have stage characteristics in the embryonic period, and myofiber morphology is a significant difference at different stages of embryonic development. On the 12th day of the embryo development, muscle fiber’s complete shape had not been formed, and the outline of muscle fiber was not clear (Fig. 1 A). With time, the cross-section of myofiber revealed a complete structure. The intervals between muscle bundles were gradually clear and distinct. At the same time, the structure of myofiber tended to mature (Fig. 1B- D). The muscle fiber surface of the embryo at E19 (8.01 ± 0.59 μm) was significantly larger *(P < 0.01)* than the embryo at E16 (6.27 ± 0.50 μm), and E21 (11.17 ± 0.87 μm) was significantly larger *(P < 0.01)* than E19 (Figure S1A). The cross-section area of muscle fibers presented a trend similar to diameter (Figure S1B).

### Overview of RNA-sequencing

To obtain complete and accurate mRNA transcripts of the chicken embryo, we constructed 12 cDNA libraries (E12-1, E12-2, E12-3, E16-1, E16-2, E16-3, E19-1, E19-2, E19-3, E21-1, E21-2, and E21-3) from embryo leg muscle. A total of 1,337,535,812 raw reads were generated from 12 cDNA libraries. Clean reads totaling 1,334,509,224 were obtained after filtering out adaptor, N ratio greater than 10 % reads, base reads, and low-quality reads. The percentage of clean reads for each duplicate was greater than 99 % (Supplementary Table 1). With an error rate of 0.1 %, more than 94 % of bases were accurately identified (Supplementary Table 2). A comparison of reference area statistics showed that approximately 50-60 % of reads match the exon region (Supplementary Table 3). Mapping the sequence of the chicken’s reference genome, which was about 5 % did not match the genome sequence (Supplementary Table 4). About 80 % of transcripts had high gene coverage (Supplementary Figure S2). All samples were distributed randomly and uniformly, and the number of genes showed trends to saturation (Supplementary Figure S3).

**Fig. 2:**
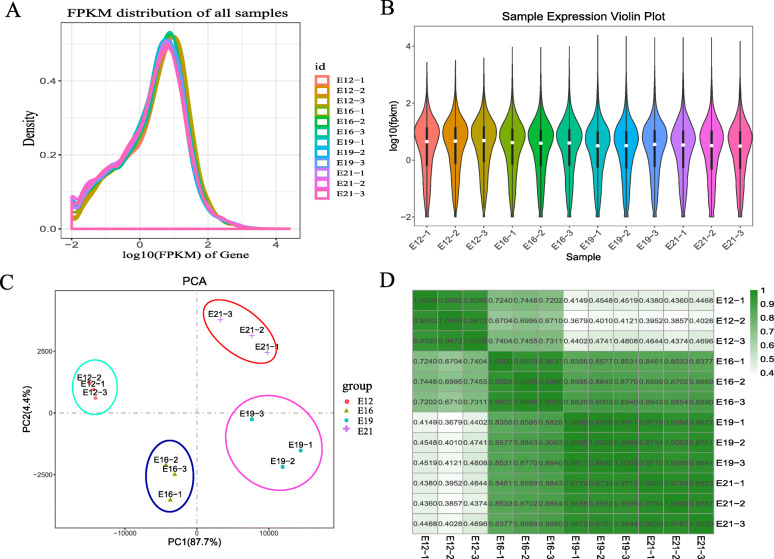
mRNA expression analysis. (**A**) The density distribution of mRNAs was according to log10 (FPKM); (**B**) The 12 Samples expression (E12-1, E12-2, E12-3, E16-1, E16-2, E16-3, E19-1, E19-2, E19-3, E21-1, E21-2, E21-3) violin plot, which was replaced by log10 (FPKM). (**C**) The PCA distribution of 12 samples; (**D**) The sample relationship cluster analysis

Using RNA-seq, 16,330 mRNAs transcripts were detected, including 109 novel mRNAs transcripts. Transcript expression was presented by FPKM (Fragments per kilo-base of exon per million fragments mapped) value. The FPKM distribution of mRNAs is shown in Fig. 2A, whereas the expression of different samples is shown as a violin chart (Fig. 2B). To effectively find the most “main” element and structure in the data, the complex sample composition relationship was reflected on the two characteristic values of the horizontal and vertical coordinates. This aided in exploring the distance relationship between samples. The 12 samples were divided into four parts, which showed satisfactory repeatability (Fig. 2C). Then, we established a relationship cluster diagram to reflect the relationship between samples (Fig. 2D) intuitively. Sequences showed a reliable clustering effect, which ensured the veracity of the subsequent analysis.

### Analysis of differentially expressed genes (DEGs)

**Fig. 3:**
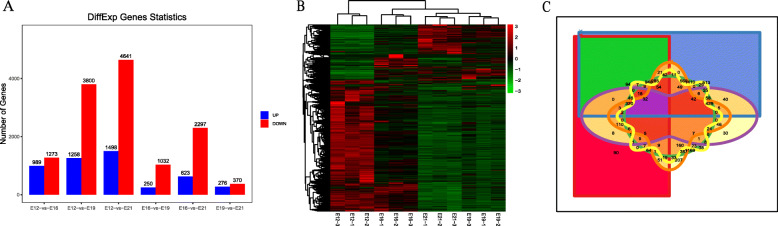
The differential expression analysis of mRNAs. (**A**) Differential gene statistics at different time points; (**B**) Differential gene cluster analysis; (**C**) The Venn plot of DEGs

We used *FDR* < 0.05 and Fold Change > 2 as the criteria to screen for differential genes by comparing pairwise differences at four time points during embryo muscle development. A total of 7,572 differentially expressed mRNAs were identified, including 2,262 in E12vsE16, 5,058 in E12vsE19, 6139 in E12vsE21, 1,282 in E16vsE19, 2,920 in E16vsE21, and 646 in E19vsE21. And the number of DEGs at different time points was summarized in Fig. 3A. Through cluster analysis, we further revealed the differential expression of genes in different periods (Fig. 3B). To identify genes that play a key role in muscle development throughout the embryonic period, we performed Venn on genes at different stages. A total of 32 key genes were found in the intersection, generated from the Venn of DEGs (Fig. 3C),and the expression of 32 key genes was shown in Tabel S6.

### Sample time series analysis of DEGs

**Fig. 4:**
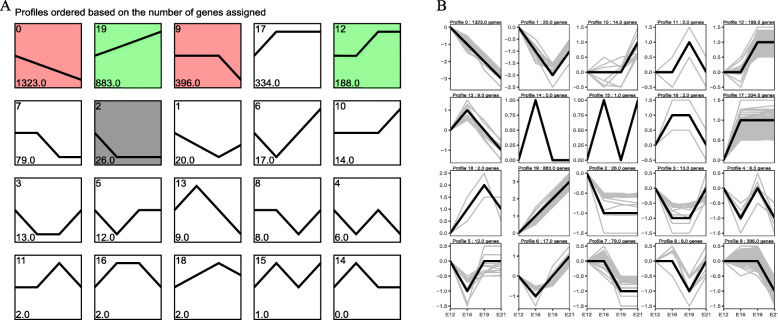
The sample time series analysis of DEGs. (**A**) Distribution trend of differential gene expression, color means significant difference (*P < 0.05*), gray means not significant (*P > 0.05*); (**B**) The time series line of differential gene expression

To comprehensively reveal muscle development status at different time points, we analyzed the expression trend of differential genes and selected more biologically meaningful target genes with *P < 0.05* as the screening condition(Gene expression was expressed as FPKM, and log_2_ normalization was performed according to the expression of the first sample). Differential genes were enriched into 20 trends, of which 4 trends appeared significant *(P < 0.05*) (Fig. 4A). The time-series line of differential gene expression is shown in Fig. 4B. The overall gene expression trend was classified as either rising or falling. The significantly enriched trends included 3 down-regulation and 2 up-regulation trends. A total of 1,745 DEGs were significantly enriched in down-regulation trends (Profile 0, profile 2, and profile 9), whereas 1,071 DEGs were enriched in up-regulation trends (profile 12 and 19). These findings effectively revealed the gene expression status of muscle development in the middle and late embryo stages.

### Functional annotation of DEGs with significant enrichment trends

**Fig. 5:**
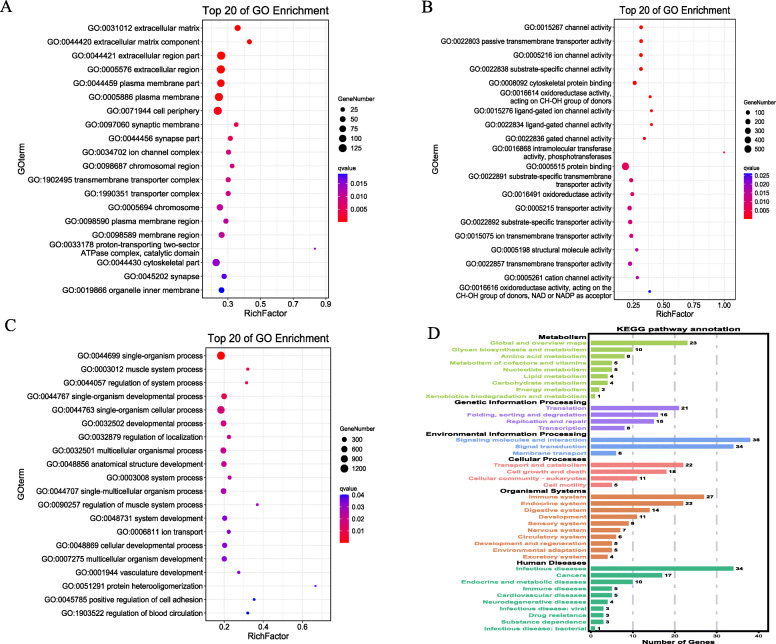
Functional analysis of significantly enriched trends. (**A**) The top 20 significance terms of Cellular Component; (**B**) The top 20 significance terms of Molecular Function; (**C**) The top 20 significance terms of Biological Process; **D** the top 20 significance terms of KEGG enrichment

Using the GO enrichment analysis, we explored the function of the target genes. The top 20 enrichment terms in the three sections (Cellular Component, Molecular Function, Biological Process) were displayed in Fig. 5A-C. Cellular components contained 65 significance terms (*P* < 0.05), such as extracellular matrix,extracellular matrix component,extracellular region part,and extracellular region. In total, 88 terms were significant enriched in molecular function, for example, channel activity,passive transmembrane transporter activity,cytoskeletal protein binding, and protein binding. And biological processes involved 453 significance terms; the top 5 terms included single-organism process,muscle system process,regulation of system process,single-organism developmental process, and single-organism cellular process.

The KEGG enrichment analysis of the DEGs was shown in Fig. 5D, including the top 20 KEGG pathways. A total of 177 KEGG pathway terms were enriched, including 19 significant terms, for instance, ECM-receptor interaction, Adrenergic signaling in cardiomyocytes, and Insulin signaling pathway. Numerous muscle development pathways were reported, including the Wnt signaling pathway (*P < 0.05*), MAPK signaling pathway, TGF-beta signaling pathway,PI3K-Akt signaling pathway and mTOR signaling pathway.

We comprehensively analyzed 32 genes selected for development at different times to identify the key mechanism by which they contribute to development. Multiple genes were enriched in muscle development-related pathways, including regulation of muscle system process, regulation of muscle contraction, and muscle system process. Furthermore, KEGG pathway analysis of 32 genes revealed that eight genes were enriched in 10 pathways, among which H2A was found to be associated with three biological pathways (Table 1).

**Table 1:** 32 key genes KEGG pathways

Pathway	Pathway ID	K_ id	Differentially expressed genes
Primary immunodeficiency	ko05340	K03648	UNG, UDG; uracil-DNA glycosylase
MicroRNAs in cancer	ko05206	K04381	STMN1; stathmin
Homologous recombination	ko03440	K10877	RAD54B; DNA repair and recombination protein RAD54B
Estrogen signaling pathway	ko04915	K09571	FKBP4_5; FK506-binding protein 4/5
Base excision repair	ko03410	K03648	UNG, UDG; uracil-DNA glycosylase
p53 signaling pathway	ko04115	K10129	GTSE1, B99; G-2 and S-phase expressed protein 1
Alcoholism	ko05034	K11251	H2A; histone H2A
Necroptosis	ko04217	K11251	H2A; histone H2A
Systemic lupus erythematosus	ko05322	K11251	H2A; histone H2A
MAPK signaling pathway	ko04010	K04381	STMN1; stathmin

### Validation of candidate genes

**Fig. 6:**
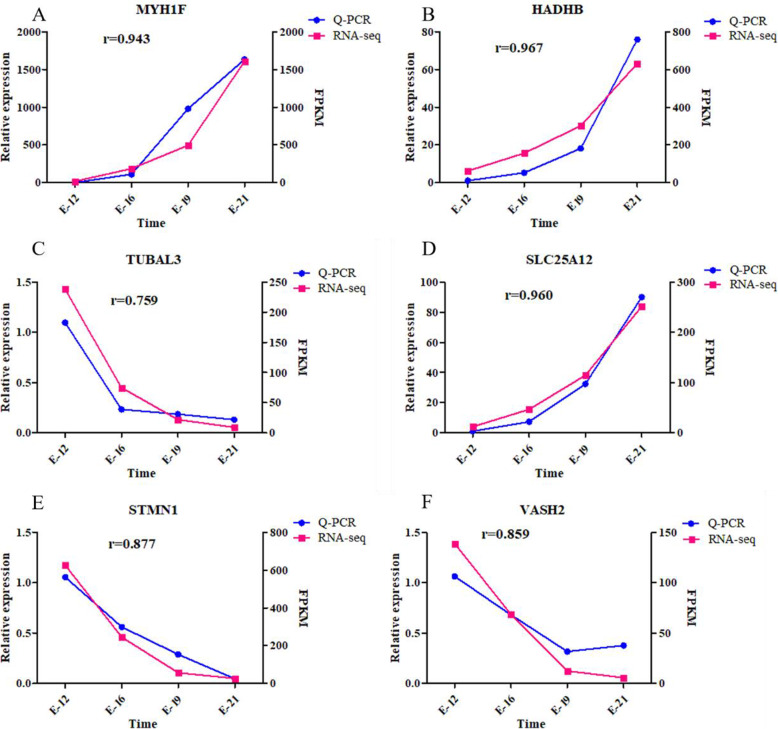
The validation of candidate genes. **A**. MYH1F; **B**. HADHB; **C**. TUBL3; **D**. SLC25A12; **E**. STMN1; **F**. VASH2. Blue means Q-PCR, red means RNA-seq and r means correlation coefficient. ACTB and GAPDH were used as the reference gene for Q-PCR, RNA-seq relative expression was represent by FPKM

To reveal the key genes associated with embryonic muscle development, we screened several genes with higher expression levels among the 32 key differentially expressed genes, including *MYH1F*, *SLC25A12* (up-regulation), *STMN1*, *VASH2*, and *TUBAL3* (down-regulation). Similarly, *HADHB* was picked out from the 109 novel genes. Upon conducting RT-qPCR verification on the selected differential genes, we generated consistent findings as with RNA-seq. Then, the candidate genes were verified via RT-qPCR (Fig. 6A-F). Notably, similar results were reported as those obtained through sequencing, which confirmed the reliability of the sequencing data.

## Discussion

Muscle development mainly occurs in two stages, the embryonic stage and the postnatal period. Among them, muscle fibers are formed in the embryonic stage,andthe number of muscle fibers remains unchanged after birth. Herein, through histological muscle analysis, we found apparent differences in the muscle of chicken embryos. Notably, on the 12th day of embryonic development, muscle fibers were yet to be formed (in the stage of fusion of multinucleated myotubes to form muscle fibers), which was similar to the formation time of intact muscle fibers in many local chicken breeds but took longer than fast-large broiler breeds [16]. A wealth of studies had shown that the embryonic period is a critical period for muscle development, during which the expression of muscle development-related genes was most active [17, 18]. To elucidate the specific variation of muscle development, we used the transcriptome analysis to explore the gene regulation network. Of note, 12 cDNA libraries at four different time points were established for transcriptome sequencing.

Muscle development is a dynamic biological process reflected in the differential expression of genes at different time points.A large number of studies related to muscle development have explained the regulatory mechanism of muscle development at different times. Transcriptome sequencing of 16-week-old Bian chickens with varying growth rates revealed 108 differential genes; 17 were up-regulated, whereas 91 were down-regulated, and which were significantly enriched in three pathways: Adrenergic signaling in cardiomyocytes, Cardiac muscle contraction and Tight junction[19]. Moreover, 364, 219, and 111 differentially expressed genes (F8FvsF4F, F8SvsF4S, and M8SvsM4S) were detected for the three comparison groups, respectively, which used the 4-week old and 8-week old males and female of Jinghai yellow chicken with different growth rates. At the same time, the ECM–receptor interaction and focal adhesion were significantly enriched in KEGG pathway analysis with differentially expressed genes [12]. In addition, transcriptome sequencing of breast muscle of Jinghai Huang chicken at different times (M4F, M8Fand M12F) was conducted to screen out 3,903 DEGs, and it was analyzed that *RAC2* may affect the growth of chickens by regulating the PAKs/MAPK8 pathway[20]. In the later stage of embryonic development, the number of differentially expressed genes decreased sharply. A similar gene expression pattern which we employed to assess muscle development after birth [17]. These findings collectively provide evidence that the embryonic stage is the critical point of muscle development and the most extensive time of gene expression. Moreover, it gives a theoretical basis for the number of muscle fibers left within a range from late embryonic development until after birth. Muscle development is variety-specific, and there are significant differences in muscle gene expression between different varieties. In a previous report, 8,398 DEGs were found among Roman, White Broiler, and Daheng chickens via transcriptome analysis,Similarly, these differential genes are also significantly enriched in the extracellular matrix-receptor interaction, MAPK signaling pathway, and focal adhesion [1]. Variable splicing of genes also regulates muscle development in different chicken breeds [21]. This is the basis for the selection and breeding of local, high-quality varieties.

Muscle development related genes have unique regulatory effects at different stages of development. Meanwhile, there are significant change in gene expression at different developmental times. In the present analysis, a total of 7,572 differentially expressed genes were detected, including 32 stage key differentially expressed genes and 59 novel genes. By comparing the gene expression at different times, the gene expression on the 12th day of embryonic was significantly different from other time points; this further validated the data for muscle sections. The 12th day of embryonic has a different expression pattern from the other three points, which is related to the stage of muscle development. The results showed that the expression of muscle development-related genes affected the development of muscle fibers, and the active level of gene expression reflected the progress of muscle development. Besides, we used the time series analysis to characterize gene expression and reveal the law of muscle development at different times. Differentially expressed genes are mainly enriched in a downward trend, implying that genes related to muscle development are highly expressed before myofiber formation and gradually decrease with the integrity of muscle fiber morphology and structure. Functional enrichment analysis was performed for different genes in the same trend to identify their involvement in the same biological process. When we performed the GO and KEGG analysis to the downward trend, numerous development-related terms were significantly enriched, including cell cycle, Wnt signaling pathway, and the extracellular matrix (ECM) receptor interaction. Of note, the cell cycle is an essential process of life activities, involving the entire process of muscle development. The development cycle of muscle cells changes to achieve muscle fiber thickening and growth. The extracellular matrix mainly includes a few insoluble proteins, such as collagen. Numerous studies have shown that the extracellular matrix is involved in cell proliferation, differentiation, and tissue structure maintenance, and it acts as a scaffold for cells and tissues[22]. Early development of skeletal muscle cells is wrapped in an extracellular matrix, promoting embryonic formation through the interaction between cells and the extracellular matrix [23]. Additionally, the interaction between the two potentially alleviates cell senescence caused by changes in the extracellular matrix. The Wnt signaling pathway plays a key role in early embryo development [24], and changes in the expression of pathway-related genes will impact body functions. *KLF5* was found to be enriched in the Wnt signaling pathway. Besides, *KLF5* knockdown causes muscle atrophy in chickens [25]. At the same time, *MyoF* activates the Wnt/beta-catenin signaling pathway to regulate the expression of atrophy-related factors to rescue muscle atrophy [26].

Moreover, a comparative analysis of different times combined with the time series analysis of standard screening revealed multiple-stage differential genes, including *MYH1F*, *SLC25A12, STMN1*, *VASH2*, *TUBAL3*, and *HADHB*. *MYH1F* is a member of the MYH (myosin heavy chain) gene family. As one of the key members that regulate muscle development, the MYH family can affect muscle production and repair post-injury, mainly by regulating cell proliferation and differentiation. Elsewhere, multiple gene isoforms were found to be highly expressed in human muscle tissue [27], and *MYH15* was differentially expressed in three chicken breeds [1]. *MyHC* is the most widely researched member, and numerous reports have confirmed that it can participate in early growth regulation and late growth regulation. As a marker gene for myoblast differentiation to form multinucleated myotubes, the expression level of MyHC reflects the development status of muscle fibers [28]. In the present study, we confirmed that the expression quantity of MYH1F increased gradually with time, an implication that MYH1F mainly played a role in the growth of mature muscle fibers.

Solute carriers (SLC) are transmembrane transport carriers for amino acids and other substances. Most of them are distributed on the cell membrane, and a small portion is localized in the mitochondria. Notably, five solute carrier superfamily genes (*SLC6A9*, *SLC38A4*, *SLC22A5*, *SLC35F3*, and *SLC16A3)* were involved in melanin deposition in chicken muscles [29]. The SLC25 family is located on the mitochondria and can participate in the development of the nervous system [30]. Many solute carrier family members were detected in this study, especially the SLC25A family-related genes as a representative, among which *SLC25A4* and *SLC25A12* were differentially expressed in embryonic muscle tissue. This observation has not been described in previous studies. Simultaneously, *HADHB* is related to mitochondrial function and fatty acid metabolism. *HADHA* and *HADHB* cause mitochondrial trifunctional protein deficiency (MTPD), leading to peroneal muscular atrophy [31, 32]. Whether SLC families involved in muscle development by regulating mitochondrial function remains elusive.

*STMN1* is widely involved in tumorigenesis, tissue development, and maturation[14, 33]. In a previous study, a comparative analysis of transcriptome sequencing during skeletal muscle development of pig breeds with different muscle growth rates and degrees of hypertrophy showed that *STMN1* participated in later myogenesis and contributed to more myofibers [14]. Moreover, *STMN1* was highly expressed in muscle tissue in the early stage of embryonic development and significantly affected the proliferation of C2C12 cells [34]. Similarly, high expression of *STMN1* has been reported to involve the proliferation, migration, and invasion of various cancer cells, among them were lung cancer and colorectal cancer[33, 35]. For instance, *STMN1* was highly expressed in various tumor cells and was found associated with the clinical manifestations and malignant behavior of tumors. Thus, *STMN1* could be used as a target and a marker for the prognosis of tumor treatment. [36–38]. The highly expressed *STMN1* during the embryonic period and after birth had completely different physiological effects, which provided more ideas for the study of *STMN1*. In this study, qRT-PCR and sequence analyses verified that the expression of *STMN1* in embryonic muscle tissue over time has a downward trend, indicating that it might play a huge role in early embryonic development.

Besides, there are two additional genes related to muscle development at different stages of embryos, including *VASH2* and *TUBAL3*. Among them, VASH2 was belonged to the Vasohibin family, which is involved in angiogenesis [39, 40] and verified as a biomarker for the esophageal squamous cell carcinoma [41]. As a significant component of microtubules, tubulin is involved in various biological processes through posttranslational modification, including early embryo development, cytoskeletal maintenance, and tuberculosis and tumor-related diseases [42, 43]. However, the mechanisms by which these genes affect embryo muscle development are unknown, which necessitates future exploration.

## Conclusions

The present study explored the embryonic muscle development of the Chengkou Mountain Chicken through transcriptome analysis. Upon conducting differential expression genes analysis, 6,726 DEGs were identified, and lots of DEGs were enriched in down-regulation trends by time series analysis. Through transcription level analysis of embryonic muscle development, the gene regulation mechanism in myogenesis has extensively been elucidated. Validation of key genes for embryonic development and preliminary explanation of their role in muscle development. These results provide a theoretical basis for poultry breeding in the future.

## Methods

### Chicken embryo incubation and tissue collection

Chengkou Mountain Chicken Breeding Eggs were purchased from Chongqing Xuanpeng Agricultural Development Co. Ltd Chongqing, China. Eggs were incubated at 37.8 ^o^C and 55 % humidity. A total of 24 embryonic chickens from 4 different stages (the 12, 16, 19, and 21 embryonic ages)were used in this study. The embryos were euthanized by cervical dislocation and the leg muscle was colleted from similar sampling sites. 12 samples were stored in the RNA protection solution(QIAGEN) at -80 ^o^C for further use (RNA sequencing). At the same time, another 12 samples were used for histological observation, fixed in 4 % paraformaldehyde and stored at 4^o^C.Three individuals in each group were used as biological replicates.

### Chicken embryo muscle histomorphology

The histological characteristics of chicken embryo muscles were evaluated by routinely embedding the samples in paraffin, sectioning, hematoxylin and eosin staining. A microscope(Olympus IX53, Japan) was used to visualize histological muscle micromorphology, and images were collected by microphotographic system(Olympus DP71, Japan), each picture randomly selected 30 myofibers.

### Constructing cDNA library and sequence data analysis

Total RNA was extracted by Trizol reagent (TaKaRa, Dalian, China) from chicken embryo leg muscle at four stages, following the manufacturer’s protocol. The quality and purity of total RNA were analyzed via Nucleic Acid tester and gel electrophoresis. rRNA was removed from the total RNA(Epicentre, USA). To sequence, the Illumina HiSeqTM 4000 was used, samples were shipped to GENE DENOVO Biotechnology co. LTD (Guangzhou, China). To ensure quality, the original data was filtered. The reads were filtered as follows: (1) Reads containing an adapter was removed; (2) reads with N ratio greater than 10 % were then removed; (3) we removed all reads with A bases; (4) low quality reads (the number of bases with mass value Q ≤ 20 accounts for more than 50 % of the whole read) were eliminated. The following analysis was based on high-quality sequencing. Clean reads were compared to the ribosome database of the species using the short reads alignment tool Bowtie2. Reads with ribosomes were divided and compared to ensure no mismatches. Reserved unmapped reads were used for subsequent transcriptome analysis. Using the HISAT2 software, we conducted a comparative analysis based on the chick genome. Sample expression was determined by FPKM (Reads per Kilobase of exon model per Million mapped reads), and the sample repeatability was tested via principal component analysis.

### Analysis of differentially expressed genes and hierarchical clustering

Gene expression was replaced by fragments per kilobase of transcript per million fragments mapped (FPKM). Differential expression analysis between the four stages was performed using the DESeq2 package. Genes with FDR(false discovery rate) ≤ 0.05 and *Fold Change* ≥ 2 were considered as DEGs between two stages. Simultaneously, a hierarchical cluster analysis of differentially expressed genes was conducted using the ggplot2 package (http://www.r-project.org/).

### Sample time series analysis

The STEM (Short Time-series expression Miner) software was used to analyze the differentially expressed genes at four time points, cluster, and visually show the different gene expression patterns in the embryonic stage. The minimum variation of multiple gene screening was 2, with a maximum trend number of 20. Using log_2_ (FPKM), we standardized the data, and *P < 0.05* was the screening range of a reliable trend.

### Function enrichment analysis of DEGs

Gene Ontology (GO, http://www.geneontology.org/) analysis of the differentially expressed and target genes was performed using the DAVID25 software. KEGG (Kyoto Encyclopedia of Genes and Genomes) and Reactome (https://reactome.org/) pathway analysis for differentially expressed and target genes were conducted via KOBAS v2.0 using a hypergeometric test. GSEA (Gene Set Enrichment Analysis) was used to compensate for the lack of available information on minor genes in traditional enrichment analysis. P-value < 0.05 were considered to be significantly enriched.

### Validation by real-time quantitative PCR (RT-qPCR)

Here, we selected six DEGs to verify the sequencing results via RT-qPCR. Primers were designed by Primer Premier, as shown in Supplementary Table 5. First-strand cDNA was synthesized from 1 µg total RNA using the reverse transcriptase Revert Aid (Takara) following manufacturer’s recommendations. PCR amplification was executed in reaction volumes of 10 µL that included 1 µL of cDNA, 0.6 µL of forward and reverse primers (10 µM) for each gene, 5 µL of TB green PreMix (Takara, Japan), and 3.4 µL of RNA-free double-distilled H_2_O. The cycling conditions were as follows: 95 °C for 30 s, 40 cycles of 95 °C for 5 s, and annealing temperature 60 °C for 30 s; a melt curve analysis was performed at 65°~95°.

### Statistical analysis

ACTB and GAPDH were used as housekeeping genes for qPCR, and the mean expression levels of ACTB and GAPDH were considered to be the expression levels of housekeeping genes. E12 was used to normalized the gene expression,and the relative gene expression was calculated by 2^−△△CT^ method.Data were expressed as means ± standard deviation of the mean and subjected to unpaired Student’s t-test, whereas Duncan’s Multiple Range Test was used for two-group comparisons, via SPSS 20.0 (SPSS Inc., USA). Graphics were drawn by GraphPad Prism 7 (GraphPad Software, San Diego, CA, USA). Values were considered to be statistically significant at *P < 0.05*, and *P-value ≤ 0.01* was considered extreme significant [44].

## Supplementary Information

**Additional file 1:Table S1**. Sequencing data quality control. **Table S2**. Statistics of clean reads at 4 different time points of chicken muscle embryo. **Table S3**. Comparison of reference area statistics. **Table S4**. Reference genome alignment. **Table S5**. Primer sequencing in this study. **Table S6**.The expression of 32 key genes. **Figure S1**.Muscle fiber area and diameter statistics **Figure S2**.Gene coverage of different samples. **Figure S3**. Sample randomness distribution.

## Data Availability

The raw data has been submitted to the National Center for Biotechnology Information (NCBI) Sequence Read Archive (SRA), and the accession number is PRJNA674456.

## Abbreviations

E12,16,19,21: 12, 16, 19, 21 embryonic days
RNA-seq: RNA sequencing
DEGs: Differentially expressed genes
KEGG: Kyoto Encyclopedia of Genes and Genomes
GO: Gene ontology
DAVID: Database for Annotation, Visualization andIntegrated Discovery
MyoD: Myogenic Differentiation Antigen
MAPK: Mitogen-activated protein kinase
TGF-β: Transcription growth factor beta
mTOR: Mammalian target of rapamycin
qRT-PCR: Quantitative real-time PCR
STMN1: Stathmin isoform X1
SLC25A12: Calcium-binding mitochondrial carrier protein Aralar1 isoform X1
MYH1F: Myosin, heavy chain 1 F
TUBAL3: Tubulin alpha-3 chain
HADHB: Trifunctional enzyme subunit beta, mitochondrial isoform X1
VASH2: Vasohibin-2

## References

[1] Zhang Z, Du H, Yang C, Li Q, Qiu M, Song X, Yu C, Jiang X, Liu L, Hu C (2019). Comparative transcriptome analysis reveals regulators mediating breast muscle growth and development in three chicken breeds. ANIM BIOTECHNOL.

[2] Li C, Li X, Liu Z, Ni W, Zhang X, Hazi W, Ma Q, Zhang Y, Cao Y, Qi J (2019). Identification and characterization of long non-coding RNA in prenatal and postnatal skeletal muscle of sheep. GENOMICS.

[3] Buckingham M: Gene regulatory networks and cell lineages that underlie the formation of skeletal muscle. Proceedings of the National Academy of Sciences 2017, 114(23):5830–5837. 10.1073/pnas.1610605114 PMC5468682 28584083

[4] Jin C, Ye J, Yang J, Gao C, Yan H, Li H, Wang X (2019). mTORC1 Mediates Lysine-Induced Satellite Cell Activation to Promote Skeletal Muscle Growth. CELLS-BASEL.

[5] Bismuth K, Relaix F (2010). Genetic regulation of skeletal muscle development. EXP CELL RES.

[6] Zammit PS (2017). Function of the myogenic regulatory factors Myf5, MyoD, Myogenin and MRF4 in skeletal muscle, satellite cells and regenerative myogenesis. SEMIN CELL DEV BIOL.

[7] Borensztein M, Monnier P, Court F, Louault Y, Ripoche M, Tiret L, Yao Z, Tapscott SJ, Forne T, Montarras D (2013). Myod and H19-Igf2 locus interactions are required for diaphragm formation in the mouse. DEVELOPMENT.

[8] Hernandez-Hernandez M, Garcia-Gonzalez EG, Brun CE, Rudnicki MA (2017). The myogenic regulatory factors, determinants of muscle development, cell identity and regeneration. SEMIN CELL DEV BIOL.

[9] Yamamoto M, Legendre NP, Biswas AA, Lawton A, Yamamoto S, Tajbakhsh S, Kardon G, Goldhamer DJ (2018). Loss of MyoD and Myf5 in Skeletal Muscle Stem Cells Results in Altered Myogenic Programming and Failed Regeneration. STEM CELL REP.

[10] Xu E, Zhang L, Yang H, Shen L, Feng Y, Ren M, Xiao Y (2019). Transcriptome profiling of the liver among the prenatal and postnatal stages in chickens. POULTRY SCI.

[11] Wu P, Dai G, Chen F, Chen L, Zhang T, Xie K, Wang J, Zhang G (2018). Transcriptome profile analysis of leg muscle tissues between slow- and fast-growing chickens. PLOS ONE.

[12] Wu P, Zhang X, Zhang G, Chen F, He M, Zhang T, Wang J, Xie K, Dai G (2020). Transcriptome for the breast muscle of Jinghai yellow chicken at early growth stages. PEERJ.

[13] Hu Z, Cao J, Zhang J, Ge L, Zhang H, Liu X. Skeletal Muscle Transcriptome Analysis of Hanzhong Ma Duck at Different Growth Stages Using RNA-SEq. BIOMOLECULES 2021, 11(3152). 10.3390/biom11020315 PMC7927120 33669581

[14] Zhao X, Mo D, Li A, Gong W, Xiao S, Zhang Y, Qin L, Niu Y, Guo Y, Liu X (2011). Comparative analyses by sequencing of transcriptomes during skeletal muscle development between pig breeds differing in muscle growth rate and fatness. PLOS ONE.

[15] Liu A, AO X, MA X, WANG W, WANG X, LV X, QIAO B, LI S, Xiang B (2018). Effects of feeding mode on growth and some meat quality indexes of Chengkou Mountain Chicken. Journal of Southwest University.

[16] Zou X. Morphological comparative study on the development of leg muscle and pectoralis muscle of different breeds of chickens in embryonic stage. South China Agricultural University; 2016.

[17] Liu J, Lei Q, Li F, Zhou Y, Gao J, Liu W, Han H, Cao D. Dynamic Transcriptomic Analysis of Breast Muscle Development From the Embryonic to Post-hatching Periods in Chickens. FRONT GENET 2020, 10. 10.3389/fgene.2019.01308 PMC6967404 31998367

[18] Li T, Wang S, Wu R, Zhou X, Zhu D, Zhang Y (2012). Identification of long non-protein coding RNAs in chicken skeletal muscle using next generation sequencing. GENOMICS.

[19] He M, Wu P, Chen F, Zhang B, Chen L, Zhang T, Zhang L, Li P, Wang J, Zhang G (2020). Transcriptome analysis of leg muscles in fast and slow growth Bian chickens. ANIM BIOTECHNOL.

[20] Zhang G, Wu P, Zhou K, He M, Zhang X, Qiu C, Li T, Zhang T, Xie K, Dai G (2021). Study on the transcriptome for breast muscle of chickens and the function of key gene RAC2 on fibroblasts proliferation. BMC GENOMICS.

[21] Li Z, Xu Y, Lin Y (2018). Transcriptome analyses reveal genes of alternative splicing associated with muscle development in chickens. GENE.

[22] Theocharis AD, Manou D, Karamanos NK (2019). The extracellular matrix as a multitasking player in disease. FEBS J.

[23] Thorsteinsdottir S, Deries M, Cachaco AS, Bajanca F (2011). The extracellular matrix dimension of skeletal muscle development. DEV BIOL.

[24] Wang S, Huang H, Xiang H, Gu B, Li W, Chen L, Zhang M (2019). Wnt Signaling Modulates Routes of Retinoic Acid-Induced Differentiation of Embryonic Stem Cells. STEM CELLS DEV.

[25] Zhang D, Yin H, Li J, Wang Y, Yang C, Jiang X, DU H, Liu Y. KLF5 regulates chicken skeletal muscle atrophyvia the canonical Wnt/beta-catenin signaling pathway. *EXP ANIM TOKYO* 2020. 10.1538/expanim.20-0046 PMC7677084 32641593

[26] Han S, Cui C, He H, Shen X, Chen Y, Wang Y, Li D, Zhu Q, Yin H. Myoferlin Regulates Wnt/beta-Catenin Signaling-Mediated Skeletal Muscle Development by Stabilizing Dishevelled-2 Against Autophagy. *INT J MOL SCI* 2019, 20(513020). 10.3390/ijms20205130 PMC6829482 31623157

[27] Mascarello F, Toniolo L, Cancellara P, Reggiani C, Maccatrozzo L (2016). Expression and identification of 10 sarcomeric MyHC isoforms in human skeletal muscles of different embryological origin. Diversity and similarity in mammalian species. ANN ANAT.

[28] Zhang M, Li B, Wang J, Zhang S, Li H, Ma L, Guo W, Lei C, Chen H, Lan X (2019). lnc9141-a and -b Play a Different Role in Bovine Myoblast Proliferation, Apoptosis, and Differentiation. MOL THER-NUCL ACIDS.

[29] Yu S, Wang G, Liao J, Tang M (2018). Transcriptome profile analysis identifies candidate genes for the melanin pigmentation of breast muscle in Muchuan black-boned chicken. Poult Sci.

[30] Babenko VN, Smagin DA, Galyamina AG, Kovalenko IL, Kudryavtseva NN. Altered Slc25 family gene expression as markers of mitochondrial dysfunction in brain regions under experimental mixed anxiety/depression-like disorder. BMC NEUROSCI 2018, 19(79). 10.1186/s12868-018-0480-6 PMC6288882 30537945

[31] Lu Y, Wu R, Meng L, Lv H, Liu J, Zuo Y, Zhang W, Yuan Y, Wang Z (2018). HADHB mutations cause infantile-onset axonal Charcot-Marie-Tooth disease: A report of two cases. CLIN NEUROPATHOL.

[32] Suyama T, Shimura M, Fushimi T, Kuranobu N, Ichimoto K, Matsunaga A, Takayanagi M, Murayama K (2020). Efficacy of bezafibrate in two patients with mitochondrial trifunctional protein deficiency. Molecular genetics metabolism reports.

[33] Zhang J, Fu J, Pan Y, Zhang X, Shen L: Silencing of miR-1247 by DNA methylation promoted non-small-cell lung cancer cell invasion and migration by effects of STMN1. ONCOTARGETS THER 2016, 9:7297–7307. 10.2147/OTT.S111291 PMC5138046 27942223

[34] Balogh A, Mege RM, Sobel A (1996). Growth and cell density-dependent expression of stathmin in C2 myoblasts in culture. EXP CELL RES.

[35] Bi C, Cui H, Fan H, Li L: LncRNA LINC01116 Promotes the Development of Colorectal Cancer by Targeting miR-9-5p/STMN1. ONCOTARGETS THER 2020, 13:10547–10558. 10.2147/OTT.S253532 PMC7573327 33116633

[36] Passaia BDS, Lima K, Kremer JL, Da Conceicao BB, de Paula Mariani BM, Lipreri Da Silva JC, Nogueira Zerbini MC, Barisson Villares Fragoso MC, Machado-Neto JA, Pacicco Lotfi CF: Stathmin 1 is highly expressed and associated with survival outcome in malignant adrenocortical tumours. INVEST NEW DRUG 2020, 38(3):899–908. 10.1007/s10637-019-00846-9 31441020

[37] Cao S, Zhang W, Shen P, Xu R: Low STMN1 is associated with better prognosis in Asian patients with esophageal cancers: A meta-analysis. J GASTROEN HEPATOL 2020. 10.1111/jgh.15062 32250469

[38] Hu X, Zhang H, Zheng X, Lin Z, Feng G, Chen Y, Pan Q, Ni F (2020). STMN1 and MKI67 Are Upregulated in Uterine Leiomyosarcoma and Are Potential Biomarkers for its Diagnosis. MED SCI MONITOR.

[39] Tu M, Lu C, Lv N, Wei J, Lu Z, Xi C, Chen J, Guo F, Jiang K, Li Q, et al: Vasohibin 2 promotes human luminal breast cancer angiogenesis in a non-paracrine manner via transcriptional activation of fibroblast growth factor 2 (vol 383, pg 272, 2016). CANCER LETT 2019, 444:189–190. 10.1016/j.canlet.2016.09.031 27702660

[40] Kobayashi M, Wakabayashi I, Suzuki Y, Fujiwara K, Nakayama M, Watabe T, Sato Y: Tubulin carboxypeptidase activity of vasohibin-1 inhibits angiogenesis by interfering with endocytosis and trafficking of pro-angiogenic factor receptors. ANGIOGENESIS 2020. 10.1007/s10456-020-09754-6 33052495

[41] Yamamoto M, Ozawa S, Ninomiya Y, Koyanagi K, Oguma J, Kazuno A, Hara H, Yatabe K, Kajiwara H, Nakamura N (2020). Plasma vasohibin-1 and vasohibin-2 are useful biomarkers in patients with esophageal squamous cell carcinoma. ESOPHAGUS-TOKYO.

[42] Sun J, Cui K, Li ZP, Gao B, Huang B, Liu Q, Shi D. Improved early development potence of in vitro fertilization embryos by treatment with tubacin increasing acetylated tubulin of matured porcine oocytes. MECH DEVELOP 2020:103631. 10.1016/j.mod.2020.103631 32828904

[43] Rogowski K, Hached K, Crozet C, van der Laan S. Tubulin modifying enzymes as target for the treatment oftau-related diseases. PHARMACOL THERAPEUT 2020:107681. 10.1016/j.pharmthera.2020.107681 32961263

[44] Liu L, Xiao Q, Gilbert ER, Cui Z, Zhao X, Wang Y, Yin H, Li D, Zhang H, Zhu Q (2018). Whole-transcriptome analysis of atrophic ovaries in broody chickens reveals regulatory pathways associated with proliferation and apoptosis. SCI REP-UK.

