# Supplementary material for: Transcriptome analysis of embryonic muscle development in Chengkou Mountain Chicken
Source: BMC Genomics. 2021 Jun 9;22:431. doi: 10.1186/s12864-021-07740-w (PMC8191012; doi:10.1186/s12864-021-07740-w)

Supplementary material

Tabel S1 Sequencing data quality control

| Sample | Raw Datas | Clean Datas (%) | Adapter (%) | Low Quality (%) | Poly A (%) | N (%) |
| --- | --- | --- | --- | --- | --- | --- |
| E12-1 | 111138084 | 110903154(99.79%) | 49452 (0.04%) | 185478 (0.17%) | 0 (0.00%) | 0 (0.00%) |
| E12-2 | 81887432 | 81736844 (99.82%) | 31884 (0.04%) | 118704 (0.14%) | 0 (0.00%) | 0 (0.00%) |
| E12-3 | 73475458 | 73348018 (99.83%) | 25224 (0.03%) | 102216 (0.14%) | 0 (0.00%) | 0 (0.00%) |
| E16-1 | 81588884 | 81443096 (99.82%) | 26634 (0.03%) | 118302 (0.14%) | 0 (0.00%) | 852(0.00%) |
| E16-2 | 148553196 | 148217264(99.77%) | 72928 (0.05%) | 263004 (0.18%) | 0 (0.00%) | 0 (0.00%) |
| E16-3 | 129602476 | 129311716(99.78%) | 58592 (0.05%) | 232168 (0.18%) | 0 (0.00%) | 0 (0.00%) |
| E19-1 | 115473662 | 115228718(99.79%) | 53256 (0.05%) | 191688 (0.17%) | 0 (0.00%) | 0 (0.00%) |
| E19-2 | 130835344 | 130511036(99.75%) | 75888 (0.06%) | 248420 (0.19%) | 0 (0.00%) | 0 (0.00%) |
| E19-3 | 116795024 | 116559114(99.80%) | 53360 (0.05%) | 182550 (0.16%) | 0 (0.00%) | 0 (0.00%) |
| E21-1 | 90746886 | 90495818 (99.72%) | 52240 (0.06%) | 196780 (0.22%) | 0 (0.00%) | 2048(0.00%) |
| E21-2 | 143859724 | 143497618(99.75%) | 90398 (0.06%) | 271708 (0.19%) | 0 (0.00%) | 0 (0.00%) |
| E21-3 | 113579642 | 113256828(99.72%) | 84164 (0.07%) | 238650 (0.21%) | 0 (0.00%) | 0 (0.00%) |

Table S2 Statistics of clean reads at 4 different time points of chicken muscle embryo

| Sample | Raw Data(bp) | BF_Q20 (%) | BF_Q30 (%) | BF_N (%) | BF_GC (%) | Clean Data(bp) |
| --- | --- | --- | --- | --- | --- | --- |
| E12-1 | 16670712600 | 16369376179 (98.19%) | 15778459580 (94.65%) | 80126 (0.00%) | 7418552738 (44.50%) | 16539027690 |
| E12-2 | 12283114800 | 12062039721 (98.20%) | 11623537943 (94.63%) | 58570 (0.00%) | 5335917603 (43.44%) | 12192468046 |
| E12-3 | 11021318700 | 10828008658 (98.25%) | 10445457718 (94.78%) | 52718 (0.00%) | 4821916644 (43.75%) | 10933881477 |
| E16-1 | 12238332600 | 11995202940 (98.01%) | 11525840071 (94.18%) | 178914 (0.00%) | 5646637710 (46.14%) | 12119516139 |
| E16-2 | 22282979400 | 21895574481 (98.26%) | 21140462839 (94.87%) | 107225 (0.00%) | 10174430973 (45.66%) | 22083287013 |
| E16-3 | 19440371400 | 19102687098 (98.26%) | 18440843014 (94.86%) | 92847 (0.00%) | 8867771358 (45.62%) | 19287927739 |
| E19-1 | 17321049300 | 17049986003 (98.44%) | 16507817064 (95.30%) | 83394 (0.00%) | 7944467506 (45.87%) | 17176296312 |
| E19-2 | 19625301600 | 19284208118 (98.26%) | 18620658960 (94.88%) | 93659 (0.00%) | 9068964453 (46.21%) | 19432225822 |
| E19-3 | 17519253600 | 17224697285 (98.32%) | 16640239062 (94.98%) | 83476 (0.00%) | 7888244061 (45.03%) | 17369110202 |
| E21-1 | 13612032900 | 13368698410 (98.21%) | 12894998730 (94.73%) | 458456 (0.00%) | 6362052827 (46.74%) | 13459454090 |
| E21-2 | 21578958600 | 21169692812 (98.10%) | 20392956165 (94.50%) | 103383 (0.00%) | 10160445031 (47.08%) | 21335307637 |
| E21-3 | 17036946300 | 16691402277 (97.97%) | 16045116538 (94.18%) | 81130 (0.00%) | 7886297608 (46.29%) | 16820690764 |

Table S3 Comparison of reference area statistics

| sample | exon | intron | intergenic |  |
| --- | --- | --- | --- | --- |
| E12-1 | 53819240 (51.10%) | 43253127 (41.07%) | 8252195 (7.84%) | |
| E12-2 | 33029680 (42.56%) | 38453945 (49.55%) | 6115224 (7.88%) | |
| E12-3 | 30766495 (44.23%) | 33446216 (48.09%) | 5339798 (7.68%) | |
| E16-1 | 41594550 (53.65%) | 27699930 (35.73%) | 8227888 (10.61%) | |
| E16-2 | 71609233 (51.07%) | 54224380 (38.67%) | 14382595 (10.26%) | |
| E16-3 | 58979650 (48.31%) | 50320090 (41.22%) | 12774960 (10.46%) | |
| E19-1 | 54425652 (50.39%) | 39089957 (36.19%) | 14489227 (13.42%) | |
| E19-2 | 67524805 (55.01%) | 39509202 (32.19%) | 15717147 (12.80%) | |
| E19-3 | 54739534 (49.93%) | 41223524 (37.60%) | 13668790 (12.47%) | |
| E21-1 | 48288728 (57.60%) | 25308972 (30.19%) | 10231492 (12.21%) | |
| E21-2 | 77050019 (58.95%) | 35350630 (27.05%) | 18303448 (14.00%) | |
| E21-3 | 53152818 (51.92%) | 32551357 (31.79%) | 16675156 (16.29%) | |

Table S4 Alignment of reference genome

| Sample | Total | Unmapped (%) | Unique_ Mapped (%) | Multiple_ Mapped (%) | Total_ Mapped (%) |
| --- | --- | --- | --- | --- | --- |
| E12-1 | 110098734 | 4774172(4.34%) | 103518818 (94.02%) | 1805744 (1.64%) | 105324562(95.66%) |
| E12-2 | 81283742 | 3684893(4.53%) | 76525584 (94.15%) | 1073265 (1.32%) | 77598849(95.47%) |
| E12-3 | 72787674 | 3235165(4.44%) | 68424374 (94.01%) | 1128135 (1.55%) | 69552509(95.56%) |
| E16-1 | 80878246 | 3355878(4.15%) | 75258271 (93.05%) | 2264097 (2.80%) | 77522368(95.85%) |
| E16-2 | 146786694 | 6570486(4.48%) | 136725643 (93.15%) | 3490565 (2.38%) | 140216208(95.52%) |
| E16-3 | 128160934 | 6086234(4.75%) | 118883524 (92.76%) | 3191176 (2.49%) | 122074700(95.25%) |
| E19-1 | 113707428 | 5702592(5.02%) | 105229511 (92.54%) | 2775325 (2.44%) | 108004836(94.98%) |
| E19-2 | 129016962 | 6265808(4.86%) | 119112620 (92.32%) | 3638534 (2.82%) | 122751154(95.14%) |
| E19-3 | 115085998 | 5454150(4.74%) | 107226141 (93.17%) | 2405707 (2.09%) | 109631848(95.26%) |
| E21-1 | 89152220 | 5323028(5.97%) | 81273589 (91.16%) | 2555603 (2.87%) | 83829192(94.03%) |
| E21-2 | 139382784 | 8678687(6.23%) | 126071082 (90.45%) | 4633015 (3.32%) | 130704097(93.77%) |
| E21-3 | 108925286 | 6545955(6.01%) | 99465020 (91.31%) | 2914311 (2.68%) | 102379331(93.99%) |

Tabel S5 Primer sequencing in this study

| Gene | Forward primer(5’-3’) | Reverse primer(3’-5’) |
| --- | --- | --- |
| *VASH2* | GCAATGGCCGGTATGGATCACTG | AGGTCGCTCAGAGTTCGGTAAGTC |
| *MYH1F-F* | TGCCTCAGGTCACACTTTAGC | AGCTGTCCAATGTCAACCTTTCC |
| *TUBAL3-F* | TGAGATCCGAACTGGCACCTACC | AGTTGTTGGCAGCATCCTCCTTG |
| *HADHB-F* | CCACAGGCATAGGTTTGATAG | CAGTTCCATAGGGCTTGACA |
| *SLC25A12-F* | GGTTCCATTCCACTCCCTG | CCTGTAGTAATTTCTCCCGCTA |
| *STMN1* | GATCCTTGGTCCCCGCTCAAAAG | GCGTCTCTCTTCTGCTGCTTCC |
| *ACTB* | CTGTGCCCATCTATGAAGGCTA | ATTTCTCTCTCGGCTGTGGTG |
| *GAPDH* | GTAGTGAAGGCTGCTGCTGATG | CAAAGGTGGAGGAATGGCTGTC |

Tabel S6 The expression of 32 key genes.

| id | Symbol | E12-1 | E12-2 | E12-3 | E16-1 | E16-2 | E16-3 | E19-1 | E19-2 | E19-3 | E21-1 | E21-2 | E21-3 |
| --- | --- | --- | --- | --- | --- | --- | --- | --- | --- | --- | --- | --- | --- |
| ncbi_430557 | ACACB | 0.04 | 0.02 | 0.04 | 4.67 | 3.28 | 1.31 | 19.28 | 10.35 | 7.99 | 50.21 | 42.04 | 35.26 |
| ncbi_396508 | CKMT2 | 3.62 | 2.74 | 2.73 | 85.72 | 79.67 | 76.53 | 451.98 | 265.72 | 172.98 | 1319.07 | 771.44 | 324.66 |
| ncbi_769685 | LRRC30 | 0 | 0.11 | 0.1 | 1.1 | 0.76 | 2.18 | 5.28 | 10.22 | 10.24 | 17.74 | 23.21 | 12.53 |
| ncbi_415906 | PFKFB4 | 1.7 | 0.93 | 1.39 | 16.21 | 12.59 | 17.99 | 43.18 | 41.63 | 25.7 | 90.61 | 88.77 | 64 |
| ncbi_427949 | CLCN1 | 0.01 | 0.01 | 0.03 | 0.25 | 0.12 | 0.18 | 2.56 | 1.96 | 0.52 | 10.66 | 8.31 | 9.77 |
| ncbi_768566 | MYH1F | 17.59 | 14.49 | 18.88 | 81.52 | 205.78 | 267.72 | 442.03 | 619.49 | 418.7 | 965.72 | 1899.53 | 1963.93 |
| ncbi_112533510 | LOC112533510 | 1.96 | 2.63 | 1.14 | 20.55 | 16.76 | 23.26 | 63.38 | 63.09 | 39 | 127.68 | 111.12 | 111.75 |
| ncbi_101751240 | CCDC92B | 0.04 | 0.02 | 0.04 | 0.05 | 0.5 | 0.42 | 2.36 | 1.77 | 1.86 | 6.14 | 7.95 | 4.65 |
| ncbi_430280 | HSPB7 | 0.94 | 0.73 | 1.41 | 8.73 | 2.34 | 3.55 | 29.79 | 24.57 | 13.51 | 120.3 | 87.73 | 24.64 |
| ncbi_107049660 | LOC107049660 | 3.68 | 3.22 | 3.45 | 20.37 | 15.93 | 11.92 | 58.52 | 45.98 | 38.93 | 228.33 | 208.43 | 170.5 |
| ncbi_420569 | ASB4 | 0.09 | 0.12 | 0.24 | 0.7 | 0.59 | 0.7 | 7.98 | 5.31 | 2.66 | 33.02 | 30.48 | 30.26 |
| ncbi_422546 | SLC25A4 | 46.06 | 53.97 | 51.92 | 226.99 | 159.71 | 258.59 | 1017.9 | 773.83 | 583.8 | 3302.82 | 2802.09 | 3380.9 |
| ncbi_431387 | SLC25A12 | 11.42 | 11.77 | 13.54 | 44.63 | 38.51 | 56.58 | 116.45 | 131.13 | 96.4 | 340.28 | 218.59 | 196.06 |
| ncbi_422181 | PHKA1 | 2.87 | 2.56 | 2.5 | 8.34 | 6.71 | 11.43 | 27.1 | 32.13 | 17.4 | 85.82 | 52.66 | 57.1 |
| ncbi_395650 | PHOSPHO1 | 2.1 | 1.96 | 2.46 | 6.99 | 6.38 | 7.38 | 32.24 | 20.39 | 22.4 | 63.13 | 40.84 | 48.69 |
| ncbi_396356 | MYLK2 | 2.15 | 1.3 | 1.04 | 5.24 | 4.73 | 4.09 | 25.8 | 18.22 | 13.24 | 40.05 | 51.34 | 62.01 |
| ncbi_416745 | COQ8A | 49.82 | 33.26 | 35.24 | 133.24 | 112.9 | 111 | 350.26 | 200.9 | 172.67 | 739.6 | 576.1 | 472.24 |
| ncbi_421186 | FKBP5 | 1.96 | 1.57 | 3.57 | 7.39 | 7.47 | 5.05 | 28.23 | 30.46 | 26.69 | 55.41 | 62.27 | 87.43 |
| ncbi_431066 | PNPLA2 | 14.72 | 10.89 | 9.27 | 15.85 | 28.17 | 44.41 | 129.62 | 33.63 | 50.66 | 430.69 | 259.86 | 322.96 |
| ncbi_424819 | CLSTN2 | 2.98 | 2.39 | 2.52 | 1.03 | 1.27 | 1.63 | 0.58 | 0.41 | 0.47 | 0.11 | 0.15 | 0.12 |
| ncbi_415993 | CHDH | 20.59 | 31.4 | 23.71 | 16.39 | 11.99 | 9.26 | 6.35 | 6.39 | 5.12 | 1.33 | 1.49 | 1.23 |
| ncbi_421364 | VASH2 | 136.21 | 153.62 | 126.6 | 52.98 | 83.12 | 69.2 | 8.53 | 19.21 | 9.39 | 4.5 | 6.88 | 5.57 |
| ncbi_374010 | UNG | 13.7 | 8.99 | 13.01 | 4.91 | 5.35 | 6.73 | 0.17 | 1.33 | 1.99 | 0 | 0 | 0 |
| ncbi_396252 | CDK1 | 93.54 | 117.12 | 140.51 | 49.41 | 48.59 | 50 | 8.7 | 11.9 | 10.61 | 5.29 | 5.58 | 3.91 |
| id | Symbol | E12-1 | E12-2 | E12-3 | E16-1 | E16-2 | E16-3 | E19-1 | E19-2 | E19-3 | E21-1 | E21-2 | E21-3 |
| ncbi_423007 | KNL1 | 15.34 | 17.64 | 19.61 | 7.24 | 7.49 | 7.27 | 1.87 | 2.36 | 2.51 | 0.73 | 1.15 | 0.99 |
| ncbi_418882 | CKAP2 | 39.03 | 47.4 | 54.99 | 15.59 | 21.57 | 18.59 | 5.15 | 8.65 | 4.93 | 2.26 | 3.19 | 2.66 |
| ncbi_396057 | STMN1 | 555.56 | 672.53 | 648.87 | 246.95 | 266.43 | 221.82 | 42.53 | 64.01 | 62.66 | 22.22 | 30.83 | 26.81 |
| ncbi_776499 | DCLK3 | 5.35 | 6.75 | 6.99 | 3.42 | 1.67 | 2.14 | 0.99 | 0.89 | 0.43 | 0.21 | 0.2 | 0.23 |
| ncbi_373983 | BRCA1 | 14.74 | 18.55 | 20.3 | 5.94 | 6.66 | 7.09 | 1.47 | 1.6 | 2.42 | 0.45 | 0.89 | 0.92 |
| ncbi_770690 | PODXL2 | 16.5 | 15.28 | 16.19 | 3.91 | 6.97 | 5.77 | 1.14 | 0.89 | 0.99 | 2.69 | 1.88 | 1.7 |
| ncbi_416694 | TUBAL3 | 267.03 | 207.77 | 239.11 | 74.49 | 65.74 | 84.41 | 23.28 | 21.44 | 21.42 | 8.15 | 10.98 | 8.29 |
| ncbi_395449 | RAD54B | 9.55 | 11.33 | 11.39 | 3.02 | 2.91 | 3.73 | 0.81 | 1.27 | 0.92 | 0.32 | 0.28 | 0.29 |

Figure legends

Figure S1. Muscle fiber area and diameter statistics. (A) Muscle diameter of three stages myofiber; (B) muscle cross sectional area (CSA) of three stages myofiber. Results were expressed as mean±standard deviation(n=30);All P values werecalculated using two-tailed unpaired Student’s t test. ****P<0.01*.

Figure S3. Gene coverage of different samples. Sample gene coverage distribution (1) E12-1; (2) E12-2; (3) E12-3; (4) E16-1; (5) E16-2; (6); E16-3; (7) E19-1; (8) E19-2; (9) E19-3; (10) E21-1; (11) E21-2; (12) E21-3.Replace different gene coverage ratios with different colors.

Figure S3. Sample randomness distribution. Random distribution of samples in different periods (a) E12-1; (b) E12-2; (c) E12-3; (d) E16-1; (e) E16-2; (f); E16-3; (g) E19-1; (h) E19-2; (i) E19-3; (j) E21-1; (k) E21-2; (l) E21-3.

Figure S1


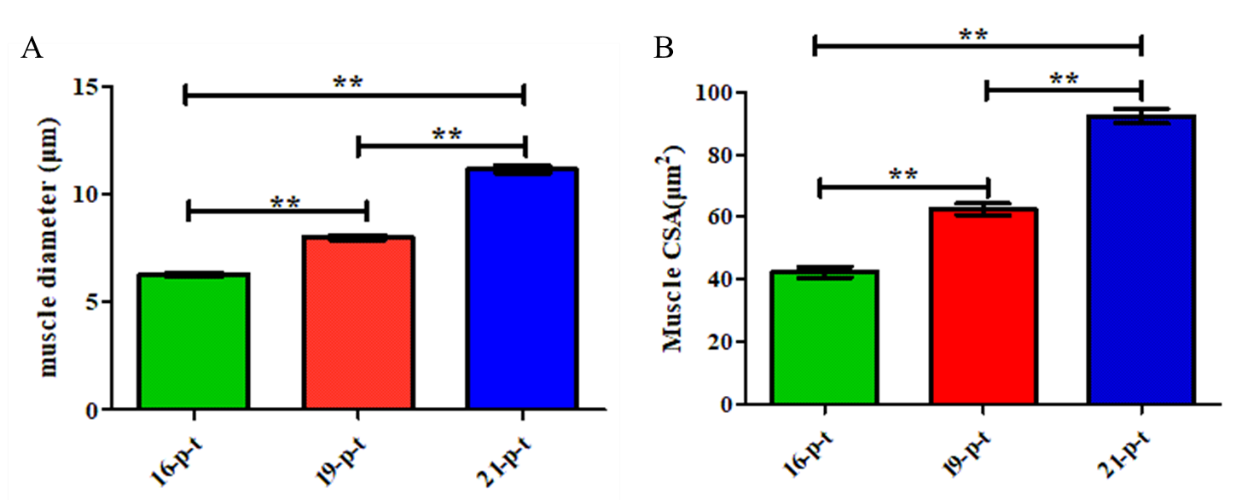


Figure S2


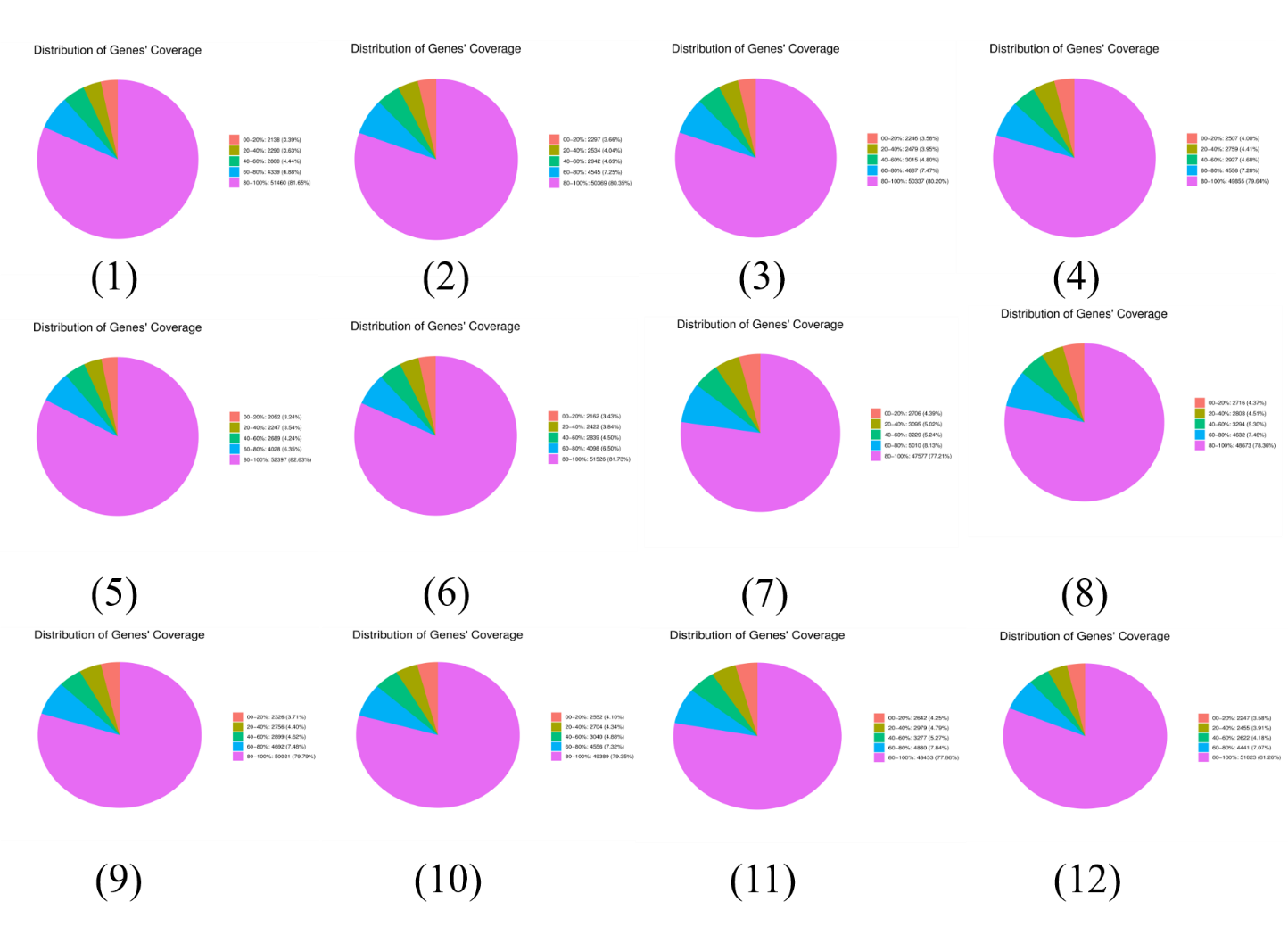


Figure S3


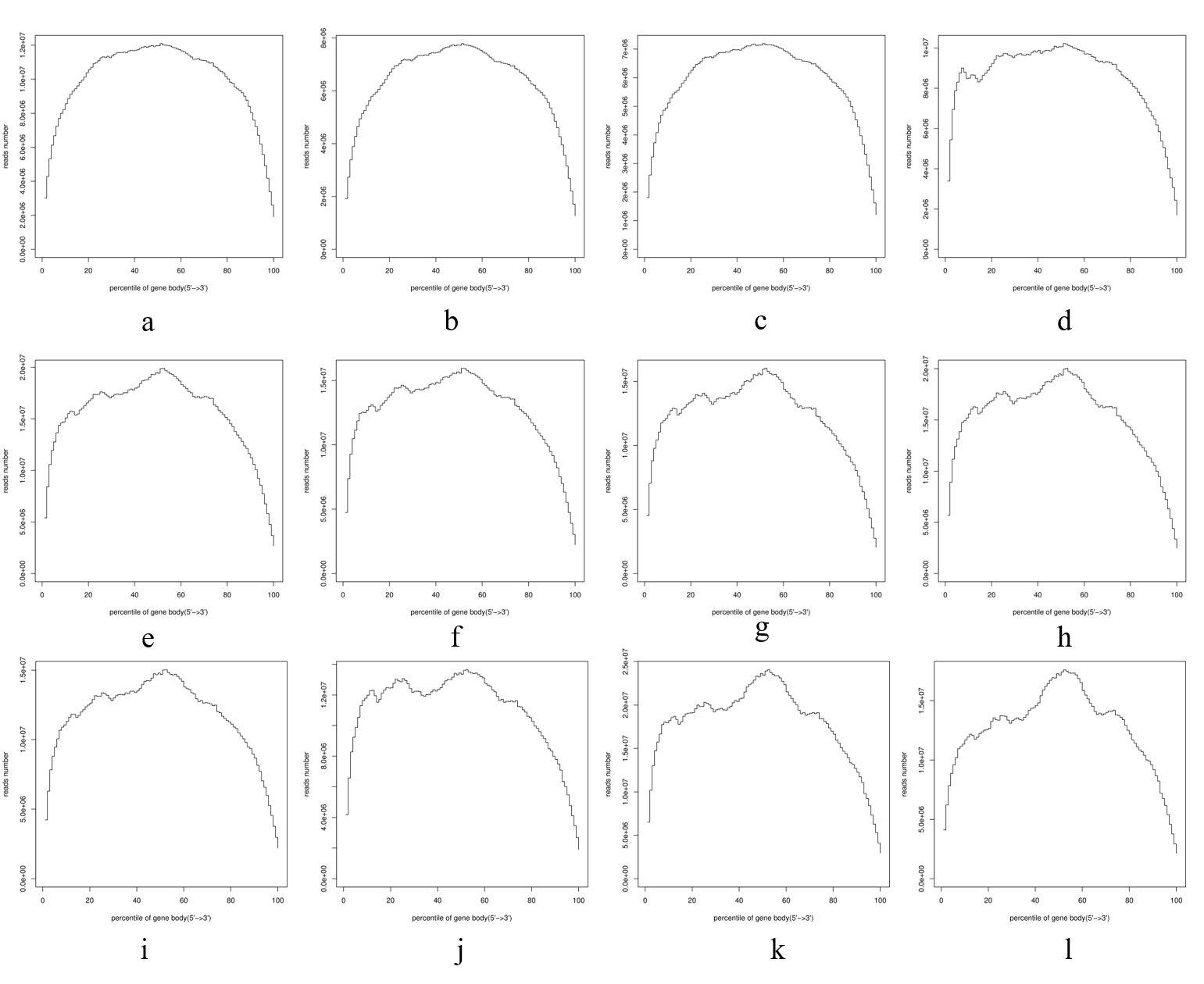

Supplement: Supplementary file 1 — Additional file 1:Table S1. Sequencing data quality control. Table S2. Statistics of clean reads at 4 different time points of chicken muscle embryo. Table S3. Comparison of reference area statistics. Table S4. Reference genome alignment. Table S5. Primer sequencing in this study. Table S6.The expression of 32 key genes. Figure S1.Muscle fiber area and diameter statistics Figure S2.Gene coverage of different samples. Figure S3. Sample randomness distribution. [file 12864_2021_7740_MOESM1_ESM.docx]
